# Role of Ginkgo biloba extract as an adjunctive treatment of elderly patients with depression and on the expression of serum S100B

**DOI:** 10.1097/MD.0000000000012421

**Published:** 2018-09-28

**Authors:** Chun-Xiao Dai, Chang-Chun Hu, Yu-Shan Shang, Jian Xie

**Affiliations:** Department of Clinical Psychology, Affiliated Hangzhou First People's Hospital, Zhejiang University School of Medicine, Hangzhou, PR China.

**Keywords:** depression, effect, elderly, ginkgo biloba extract, HAMA, HAMD, serum S100B, WCST

## Abstract

**Objective:**

To explore the effect of ginkgo biloba extract (EGb) as an adjunctive treatment of elderly patients with depression and the effect on the expression of serum S100B.

**Methods:**

136 elderly patients with depression were divided into EGb +  citalopram (Cit) group and Cit group equally. Efficacy was evaluated by Hamilton Depression Rating Scale (HAMD). Wisconsin Card Classification Test (WCST) was used to evaluate cognitive function. Serum S100B expression was measured with ELISA. The relationship of S100B with HAMD, Hamilton Anxiety Scale (HAMA) score, and WCST results was evaluated subsequently.

**Results:**

The time of onset of efficacy was significantly shorter in EGb + Cit group. There were significant differences in HAMD and HAMA scores after treatment than before treatment between groups (all *P* < .05). After treatment, total number of WCST test, the number of continuous errors and non-persistent errors in both groups were less than those before treatment. The correct number and classifications number were increased than before treatment. In EGb + Cit group, correct numbers and classifications were increased, and the number of persistent errors was decreased. After treatment, S100B level was decreased, and S100B levels change in EGb + Cit group was greater than in Cit group. Serum S100B level was positively correlated with HAMD and HAMA scores before treatment and positively correlated with persistent errors number in WCST.

**Conclusion:**

EGb, as an adjunctive treatment, can effectively improve depressive symptoms and reduce expression of serum S100B, which is a marker of brain injury, suggesting that EGb restores neurologic function during the treatment of depression in elderly patients and S100B participates in the therapeutic mechanism. EGb combined with depressive drugs plays synergistic role, and the time of onset of efficacy is faster than single antidepressants.

## 1 Introduction

Depression is a common and chronic disease.^[1]^ It is a group of mood disorders or affective disorders caused by various causes.^[2]^ Depression is mainly characterized by persistent low mood, loss of interest and pleasure, slow thinking, reduced willingness to act, serious suicidal thoughts and behaviors, as well as illusions and delusions in some patients.^[3,4]^ The disease has a tendency to relapse, but the etiology and pathogenesis are still unclear so far.^[5,6]^ The abnormal activation of microglia causes astrocyte glutamatergic dysfunction, contributing to vulnerability to stress; whereas the microglial antagonist could lower inflammatory process and restore the synaptic plasticity in the hippocampal CA1 region.^[7,8]^ Traditional tricyclic antidepressants are now being replaced by the new selective serotonin reuptake inhibitors (SSRIs), the most commonly administered antidepressants for the treatment of depression which have minimal psychomotor and anticholinergic effects.^[9–11]^ Citalopram (Cit) together with fluoxetine, fluvoxamine, paroxetine and sertraline belong to SSRIs.^[12]^ Cit is effective in the treatment of major depression with a substantially better tolerability profile and the absence of anticholinergic adverse effects or cardiotoxic effects.^[11,13]^ Serotonin-norepinephrine reuptake inhibitor (SNRI), such as duloxetine, is a potent inhibitor of the inactivation by neuronal reuptake of both serotonin and norepinephrine and shows good efficacy in major depressive disorder.^[14]^ Recent studies have demonstrated the advantages of adjunctive use of Chinese herbal medicine in the treatment of depression without causing any serious adverse effects.^[15,16]^

The main active ingredients of ginkgo biloba extract (EGb) are flavonoids and terpene lactone.^[17]^ The main pharmacological action of EGb for the central nervous system is to inhibit platelet activating factor (PAF), promote blood circulation, increase blood flow, improve hemorheological changes, resist free radicals, increase tolerance to hypoxia, prevent brain edema induced by trauma and toxin, and protect nerves.^[18–21]^ Recent studies have shown that EGb can improve the cognitive function of attention and memory in patients with mild to moderate Alzheimer's disease, and its curative effect is similar to that of Donepezil (cholinesterase inhibitor).^[22–24]^

S100B is mainly distributed in the astrocytes and oligodendroglia cells in the central nervous system and the peripheral nervous system in mammals, and some of the neurons.^[25,26]^ S100B is also known as a glial-derived protein.^[27]^ As an intracellular calcium receptor protein, S100B protein has extensive biological activities, such as intercellular connection, growth, cell structure, energy metabolism, movement and conduction and intracellular signal.^[28,29]^ Moreover, S100B has been reported to be implicated in regulating inflammation, learning and memory ability, and neural plasticity.^[30–32]^ Interestingly, antidepressants can reduce the concentration of S100B with the improvement of depressive symptoms.^[33]^ In both animal models and patients of depression, serum S100B levels were observed to be decreased.^[34]^ S100B is a neurotrophic factor that is involved in neuroplasticity, which is disrupted in depression; whereas, antidepressants can restore enhanced neuroplasticity in response to high serum S100B levels.^[35]^

Accordingly, the present study was carried out to explore the efficacy and adverse effects of EGb as an adjunctive treatment of elderly patients with depression and the effect on the expression of serum S100B. We hypothesized that EGb combined with Cit, as an SSRI, was superior to conventional SSRIs in the treatment of elderly patients with depression, and EGb may increase cognitive function in patients with late-onset depression.

## 2 Materials and methods

### 2.1 Study subjects

A total of 136 elderly patients with depression were selected in the Department of Psychiatry in our hospital in March 2015∼March 2017. Among the 136 cases, there were 64 males and 72 females, with a mean age of (67.3 ± 4.8) years. Inclusion criteria:

1) Age range: ≥ 60 years old;
2) In accordance with the diagnostic criteria for depressive episodes mentioned by Diagnostic and Statistical Manual of Mental Disorders issued by American Psychiatric Association (DSM-V);^[36]^ Hamilton depression scale 24 (HAMD-24) scores ≥ 21 points; 3) Patients who did not take plenty of medicines or alcohol within 2 weeks, or wasn’t addicted to alcohol or medicines (including lifelong addicts to alcohol or substance addicts or substance abusers);
3) No organic somatic diseases in the brain or, somatic diseases (including severe cardiovascular diseases, kidney diseases, liver diseases, endocrine diseases or blood diseases);
4) No schizophrenia, mania, bipolar disorder and other psychiatric disorders;
5) Patients with no allergy to EGb and Cit. If there was a history of psychotropic drugs administration, the patient needed to withdraw the drugs for 2 weeks as a cleaning period. 7) Non-illiterate patients. Included subjects were divided into 2 groups according to the odd and even number of diagnosis and treatment card number: EGb + Cit group (68 cases) and Cit group (68 cases). All the subjects agreed and signed the written informed consent, and this study was approved by the ethics committee of the hospital.

### 2.2 Therapeutic method

Patients in Cit group were treated with Cit. Concrete measures: Oral administration of Cit for patients in this group, 20 mg per day, and 1 time a day. On the basis of Cit group, EGb + Cit group was treated with EGb Tablets (Harbin HaoBo Pharmaceutical Co., Ltd.) for treatment, 19.2 mg per time, and 3 times a day. Two groups of patients were allowed to use a small amount of benzodiazepines to control insomnia and other symptoms when necessary.

### 2.3 Observation indexes and evaluation of curative effect

Before treatment and after treatment, 5 mL of fasting venous blood was collected from the 2 groups of patients at 6:30 to 8:00 in the morning. Then the blood sample was then placed at room temperature for 30 minutes, followed by centrifugation at 2500 r/m for 15 min. After this, the sample was stored at −80 °C for further usage after the separation of serum. The serum S100B levels were measured according to the instructions of the human S100B ELISA Kit (Nanjing JianCheng Biotechnology Co., Ltd., Nanjing, Jiangsu, China). Furthermore, 2 trained psychiatrists (Kappa = 0.92) evaluated the efficacy of the subjects at the beginning of treatment and at the 2, 4, 6, 8, and 12 weekends after the treatment regularly. The curative effect was evaluated by the Hamilton Depression Rating Scale (HAMD) and the Hamilton Anxiety Scale (HAMA), and the adverse effects in the treatment were evaluated by the Treatment Emergent Symptoms Scale (TESS). The Wisconsin Card Classification Test (WCST) with unified guidance was used to evaluate the cognitive function of the patients with depression. Examinations of routine blood test, routine urine test, liver function, renal function and electrocardiogram were performed once a week during the treatment.

### 2.4 Statistical analysis

SPSS21.0 (SPSS, Inc, Chicago, IL) software package was used to analyze all the data. The categorical data were expressed as the rate, percentage or the constituent ratio, and chi-square test was used for statistical analysis. The measurement data were expressed as mean ± standard deviation. Analysis of variance (ANOVA) analysis was used in comparison among multiple groups, and *t* test was used in comparison between 2 groups. Correlation analysis was performed with Pearson correlation analysis. *P* *<* .05 means that the difference was statistically significant.

## 3 Results

### 3.1 General data comparison

The 136 included subjects were divided into 2 groups of EGb + Cit group (68 cases) and Cit group (68 cases). In the 68 cases in the Cit group, there were 31 males and 37 females, with an average age of (66.82 ± 3.35) years. Meanwhile, in the 68 cases of EGb + Cit group, there were 33 males and 35 females, with a mean age of (66.48 ± 4.12) years. There was no obvious statistical difference in the gender (male/female), age, body weight, body mass index (BMI), heart rate (HR), systolic blood pressure (SBP), diastolic blood pressure (DBP) and course of disease (*P* > .05), which was shown in Table 1. The above results suggested the 2 groups were comparable.

**Table 1:**
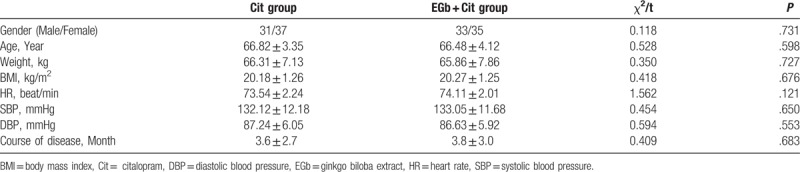
General data comparison between the 2 groups of patients.

### 3.2 Comparison of the time of onset of efficacy between groups

**Figure 1:**
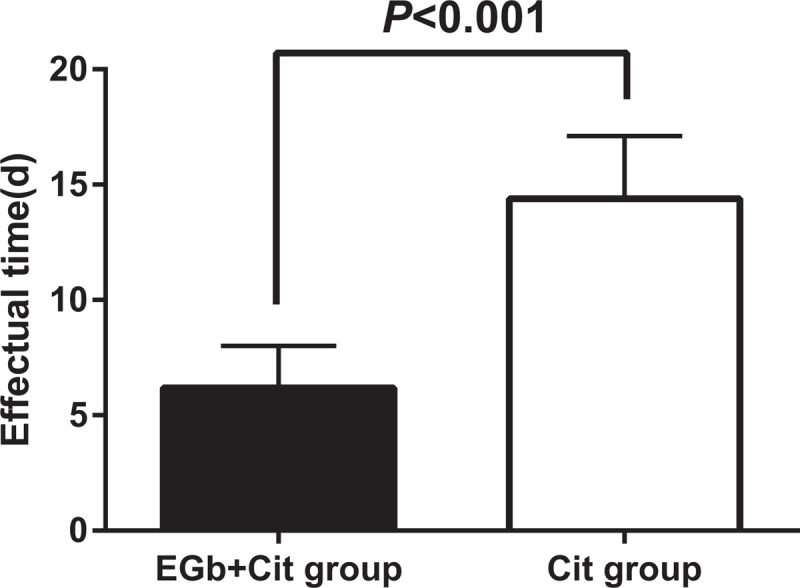
Comparison of the time of onset of efficacy between groups.

The time of onset of efficacy of EGb + Cit group and Cit group was recorded, and the results showed that the time of onset of efficacy of EGb + Cit group was 4∼15d, with a mean time of (6.2 ± 1.8)d; besides, the time of onset of efficacy of Cit group was 10∼25 d, with a mean time of (13.9 ± 3.7)d. The difference between the two groups was significant regarding the time of onset of efficacy (*P* *<* .05) (Fig. 1), as shown in Table 2. The results indicated that the effect of EGb group was faster than Cit group.

**Table 2:**
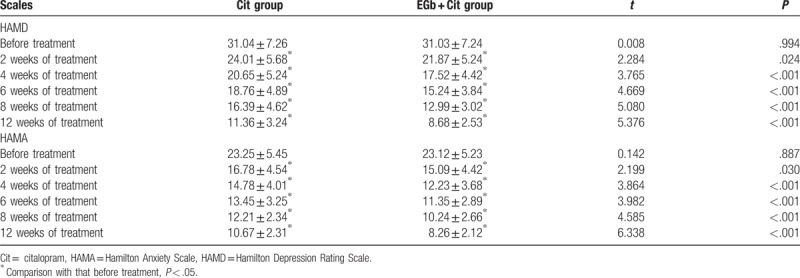
Comparison of the results of HAMD and HAMA scores between the 2 groups before and after treatment.

### 3.3 HAMD and HAMA scores comparison between groups before and after treatment

The results of HAMD and HAMA scores in EGb + Cit group and Cit group were compared before and after treatment. The results of comparative analysis showed that there was no significant difference in HAMD and HAMA scores between the 2 groups before treatment (*P* > .05). The scores of HAMD and HAMA were gradually reduced during the period of treatment, besides, there were significant differences between the two groups of HAMD and HAMA scores at the end of 2, 4, 6, 8, and 12 weeks of the treatment (*P* < .01). HAMD and HAMA scores were significantly different at the end of 2 weeks in the two groups (*P* < .05), and significant differences were found following 4, 6, 8, and 12 weeks of treatment (*P* < .01). The results suggested that the EGb + Cit group had more obvious effect, and the antidepressant and anti-anxiety effect was better than that of the Cit group.

### 3.4 Comparison of curative effect between groups

Evaluation of clinical curative effect was achieved by the current four-level assessment standard in China:^[37]^ HAMD reduction rate > 75%, complete recovery; ≥ 50%, basic recovery; ≥ 25%, improvement; < 25%, ineffective. In the 2 week treatment, the effective rate was 44.12% and 80.88% in EGb + Cit group and Cit group (*P* < .05). In the 4, 6, 8, and 12 weeks of treatment, the effective rate of Cit group was 77.94%, 85.29%, 89.70%, and 94.12%, respectively, and it was 82.35%, 88.23%, 92.65%, and 97.06% in EGb + Cit group. There was no significant difference in the comparison between groups (*P* > .05), which was shown in Table 3.

**Table 3:**
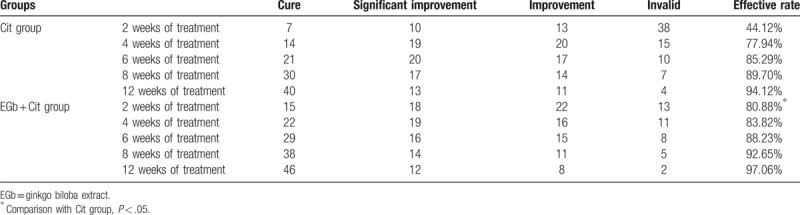
Comparison of clinical effects of 2 groups.

### 3.5 Adverse effect comparison between groups

During the treatment, the adverse effect of the 2 groups was recorded according to the TESS (Table 4). The adverse effects of the 2 groups were compared. The results showed that 19 cases had adverse effects in Cit group, the occurrence of which was 27.94%. In EGb + Cit group, 14 cases had adverse effects, the occurrence of which was 20.59%. There was no significant difference in the occurrence of adverse effects between the two groups (χ^2^ = 0.610, *P* = .435). The results showed that the combination of EGb and Cit did not increase adverse effects.

**Table 4:**
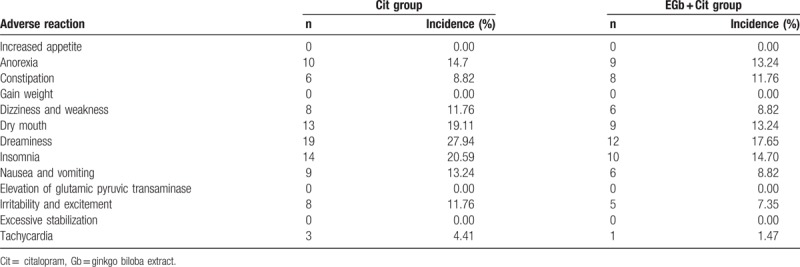
Comparison of adverse reaction between groups.

### 3.6 Comparison of WCST results before and after treatment in two groups

WCST was used to evaluate cognitive function in elderly patients with depression. Corresponding results revealed that there were no statistical differences of the total number of WCST, the correct number, the number of persistent errors, the number of non-persistent errors, and the number of classifications before treatment of the 2 groups (*P* > .05). After 12 weeks of treatment, the total number of WCST, the number of persistent errors, and the number of non-persistent errors were less in the two groups than those before the treatment (*P* < .05), while the correct number and the number of classifications were increased before the treatment (*P* < .05). The number of correct and classification in EGb + Cit group was higher than that in Cit group, and the number of persistent errors was less than that in Cit group, and the difference was statistically significant (*P* < .05). Detailed information was shown in Table 5.

**Table 5:**
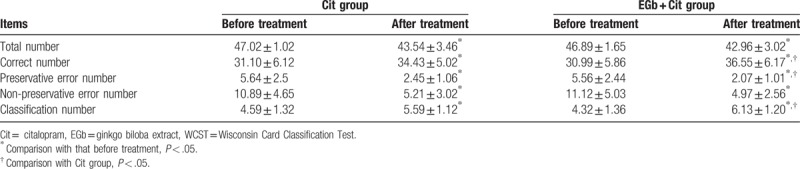
Comparison of WCST results before and after treatment in the 2 groups.

### 3.7 Detection and analysis of the level of S100B protein before and after treatment in two groups

**Figure 2:**
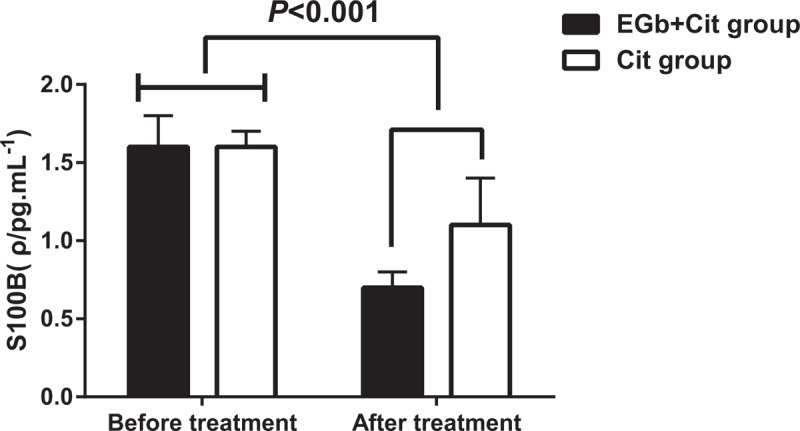
Detection and analysis of the level of S100B protein before and after treatment in 2 groups.

Serum S100B protein level in two groups of elderly patients with depression was detected by enzyme-linked immunosorbent assay before and after treatment. It was found that (Fig. 2) before treatment, there was no significant difference in the content of S100B protein between the EGb + Cit group and the Cit group (*P* > .05). After treatment, S100B level was decreased significantly in the 2 groups (*P* < .05), the change of the level of S100B in EGb + Cit group was significantly higher than that in Cit group (*P* < .05).

### 3.8 Relationship of HAMD and HAMA scores, and WCST results with serum S100B protein level

The relationship between serum S100B protein level and HAMD, HAMA, and WCST results (total number of tests, correct number, the number of persistent errors, the number of non-persistent errors, and number of classifications) was evaluated in elderly depressive patients by Pearson correlation analysis before treatment. Correlation analysis results (Table 6) showed that the level of serum S100B protein before treatment was positively correlated with the HAMD and HAMA scores (r = 0.558, *P* < .001; r = 0.582, *P* < .001), and was positively correlated with the number of persistent errors in WCST (r = 0.543, *P* < .001). However, there was no correlation between serum S100B level before treatment and the results of WCST (total number of tests, correct number, the number of persistent errors, the number of non-persistent errors, number of classifications) (*P* > .05).

**Table 6:**
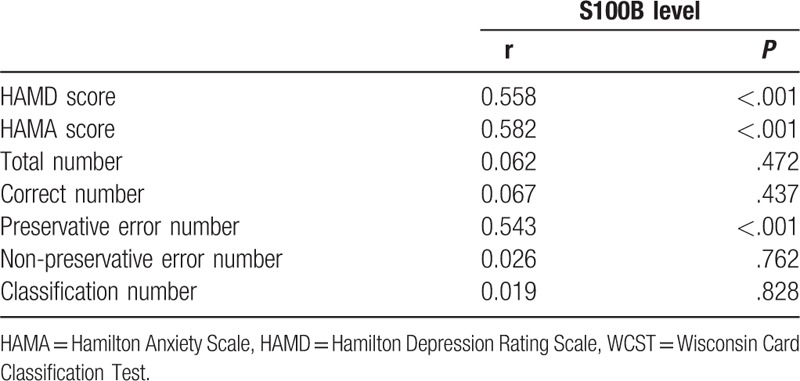
Correlation of HAMD, HAMA score, WCST results with serum S100B level.

## 4 Discussion

EGb, as a natural medicine, contains flavonoids, lactone, polyisoenenol, and other active ingredients.^[38]^ It has mild property, few toxic and adverse effects, and definite curative effects.^[39,40]^ EGb has the function of dilating blood vessels and reducing vascular resistance.^[41]^ Besides, EGb can improve myocardial function recovery, reduce the number of ventricular extrasystoles reperfusion caused by ventricular shortening of the duration of the tachycardia.^[42,43]^ In the study focusing on the role of brain circulation and blood-brain barrier, it is found that EGb can protect the brain, prevent the cerebral ischemia and improve peripheral blood circulation.^[20]^ Furthermore, its role in the central nervous system is also significant, which can be used to resist aging, improve memory, and promote neuroprotection.^[44,45]^ At the same time, it can scavenge free radicals and exert antioxidant properties.^[18]^ The clinical application of EGb has been found to have significant effects on schizophrenia, depression and Alzheimer's disease.^[23,46,47]^

As a sign of damage to the central nervous system, S100B protein has always been a hot spot in clinical research, and it has a very important clinical value.^[48]^ S100B is one of the most important members of S100 family.^[49]^ It is an EF-hand calcium-binding protein with small molecular weight, which is widely distributed in various tissues.^[50]^ It can easily pass through blood-brain barrier and has many biological functions.^[51]^ In mammals, S100B is mainly located in astrocytes, oligodendrocytes and peripheral nervous system of Schwann cells, which is closely implicated in the blood-brain barrier and brain injury.^[52]^ In addition, it also exists in a small number of non-neural tissues, such as melanocytes, chondrocytes and adrenal adjoining cells.^[53]^

At present, the treatment of geriatric depression includes drug therapy, psychotherapy, electroconvulsive therapy and combined treatment of different plans.^[54,55]^ Antidepressant therapy is the most important treatment for geriatric depression.^[56]^ Kennedy et al pointed out that patients with major depressive disorders who tolerated Cit and who had partial response were more likely to benefit from adjunctive strategies compared to switching.^[57]^ For a long time, the prognosis of geriatric depression treatment at home and abroad is unsatisfactory, and most of them are pessimistic.^[58,59]^ It indicates that there may be great differences in the process of treatment observation.

The 136 included elderly patients with depression were divided into such 2 groups as EGb + Cit group and Cit group. Patients in Cit group were treated with Cit. On the basis of Cit group, EGb + Cit group was treated with EGb Tablets. The curative effect was evaluated by HAMD and HAMA, and the adverse effect was evaluated by TESS. The WCST with unified guidance was used to evaluate the cognitive function of the patients with depression. S100B expression was measured in serum of patients with ELISA. The relationship of S100B with HAMD, HAMA score, and WCST results was evaluated using Pearson correlation analysis. The results showed that he time of onset of efficacy was significantly shorter in EGb + Cit group than that in Cit group. The scores of HAMD and HAMA were gradually reduced during the period of treatment, which was significantly different at the end of 2, 4, 6, 8, and 12 weeks of the treatment. HAMD and HAMA scores were different at the end of 2 weeks, and obviously different following 4, 6, 8, and 12 weeks of treatment in both groups. In addition, the effective rates in both groups were significantly different at the end of 2 weeks of treatment, but no difference was observed after 4, 6, 8, and 12 weeks of treatment. These results suggest that EGb does have a good effect on the treatment of depression. For a long time, the dysfunction of the central 5-hydroxytryptamine (5-HT) system is considered to play a key role in the process of depression.^[60]^ 5-HT can also promote platelet aggregation and vasoconstriction in addition to the release of platelets in the periphery of the center as a neurotransmitter.^[61]^ Because of the dual role of 5-HT dysfunction in promoting depression and promoting thrombosis, 5-HT mediated platelet activation may be a common pathophysiological basis for depression and vascular diseases.^[62]^ Ginkgolide in EGb is a highly exclusive PAF receptor blocker and can antagonize abnormal platelet aggregation and thrombosis caused by PAF.^[63]^ It is thus suggested that EGb may be workable by inhibiting platelet activation in antidepressant therapy. On the other hand, the executive dysfunction associated with depression often leads to the slow, low and unstable antidepressant effects.^[64,65]^ Since EGb has neuroprotective effect and can improve the cognitive function such as attention and memory, it may also promote the improvement of depressive symptoms.

In addition, there was no significant difference in the occurrence of adverse effects between the 2 groups. It indicates that EGb combined with Cit is well tolerated, safe and suitable for elderly patients with depression. Simultaneously, after treatment, the WCST test, the number of continuous errors and the number of non-persistent errors in the 2 groups were less than those before the treatment. The correct number and the number of classifications were increased compared with those before the treatment. In EGb + Cit group, the number of correct numbers and the number of classifications were increased, and the number of persistent errors was decreased. After treatment, S100B level was decreased significantly in the 2 groups, the change of the level of S100B in EGb + Cit group was significantly greater than that in Cit group. The level of serum S100B was positively correlated with HAMD and HAMA scores before treatment, and a positive correlation was found with the number of persistent errors in WCST.

In conclusion, EGb was an effective adjunctive treatment in improving depressive symptoms and reducing the expression of S100B in serum in the treatment of elderly patients with depression. It played a synergistic role with the combination of Cit, in which the time of onset of efficacy is faster than the single use of antidepressants. EGb may be used in adjunctive therapy to increase cognitive functions in elderly patients with depression. However, there are still limitations in this study. The sample sizes were relatively small and extended investigations should be performed in the future to validate the findings. Also, long-term follow-up observation for the efficacy and adverse effects is needed. Furthermore, future studies are needed to clarify the specific molecular mechanisms associated with the function of EGb.

## Acknowledgment

We would like to acknowledge the reviewers for their helpful comments on this paper.

## Author contributions

**Conceptualization:** Chun-Xiao Dai, Jian Xie.

**Data curation:** Chun-Xiao Dai.

**Formal analysis:** Chang-Chun Hu.

**Methodology:** Yu-Shan Shang.

## References

[1] Anyfanti P Gavriilaki E Pyrpasopoulou A Depression, anxiety, and quality of life in a large cohort of patients with rheumatic diseases: common, yet undertreated. Clin Rheumatol 2016;35:733–9. 24859781 10.1007/s10067-014-2677-0

[2] Cozzolongo R Porcelli P Cariola F Serotonin gene polymorphisms and lifetime mood disorders in predicting interferon-induced depression in chronic hepatitis C. J Affect Disord 2015;183:90–7. 26001668 10.1016/j.jad.2015.04.056

[3] Aalbers S Fusar-Poli L Freeman RE Music therapy for depression. Cochrane Database Syst Rev 2017;11:CD004517. 29144545 10.1002/14651858.CD004517.pub3 PMC6486188

[4] Wang C Guo J Guo R Effect of XingPiJieYu decoction on spatial learning and memory and cAMP-PKA-CREB-BDNF pathway in rat model of depression through chronic unpredictable stress. BMC Complement Altern Med 2017;17:73. 28118829 10.1186/s12906-016-1543-9 PMC5260079

[5] Farb NA Irving JA Anderson AK A two-factor model of relapse/recurrence vulnerability in unipolar depression. J Abnorm Psychol 2015;124:38–53. 25688431 10.1037/abn0000031 PMC4332552

[6] Baldessarini RJ Lau WK Sum MY Duration of initial antidepressant treatment and subsequent relapse of major depression. J Clin Psychopharmacol 2015;35:75–6. 25502491 10.1097/JCP.0000000000000263

[7] Kim YK Na KS Role of glutamate receptors and glial cells in the pathophysiology of treatment-resistant depression. Prog Neuropsychopharmacol Biol Psychiatry 2016;70:117–26. 27046518 10.1016/j.pnpbp.2016.03.009

[8] Finkelmeyer A Nilsson J He J Altered hippocampal function in major depression despite intact structure and resting perfusion. Psychol Med 2016;46:2157–68. 27192934 10.1017/S0033291716000702

[9] Skapinakis P Bakola E Salanti G Efficacy and acceptability of selective serotonin reuptake inhibitors for the treatment of depression in Parkinson's disease: a systematic review and meta-analysis of randomized controlled trials. BMC Neurol 2010;10:49. 20565960 10.1186/1471-2377-10-49 PMC2903535

[10] Geddes JR Freemanle N Mason J WITHDRAWN: selective serotonin reuptake inhibitors (SSRIs) versus other antidepressants for depression. Cochrane Database Syst Rev 2007;CD001851. 17636689 10.1002/14651858.CD001851.pub2 PMC10759268

[11] Mathur A Sharma DK Choudhary A Efficacy and safety of citalopram versus amitriptyline in the treatment of major depression. Indian J Psychiatry 2005;47:89–93. 20711288 10.4103/0019-5545.55952 PMC2918306

[12] Baumann P Pharmacology and pharmacokinetics of citalopram and other SSRIs. Int Clin Psychopharmacol 1996;11 Suppl 1:5–11. 8732438 10.1097/00004850-199603001-00002

[13] Hyttel J Citalopram—pharmacological profile of a specific serotonin uptake inhibitor with antidepressant activity. Prog Neuropsychopharmacol Biol Psychiatry 1982;6:277–95. 6128769 10.1016/s0278-5846(82)80179-6

[14] Girardi P Pompili M Innamorati M Duloxetine in acute major depression: review of comparisons to placebo and standard antidepressants using dissimilar methods. Hum Psychopharmacol 2009;24:177–90. 19229839 10.1002/hup.1005

[15] Xie M Jiang W Yang H Efficacy and safety of the Chinese herbal medicine shuganjieyu with and without adjunctive repetitive transcranial magnetic stimulation (rTMS) for geriatric depression: a randomized controlled trial. Shanghai Arch Psychiatry 2015;27:103–10. 26120260 10.11919/j.issn.1002-0829.214151 PMC4466851

[16] Sheng CX Chen ZQ Cui HJ Is the Chinese medicinal formula Guipi Decoction () effective as an adjunctive treatment for depression? A meta-analysis of randomized controlled trials. Chin J Integr Med 2017;23:386–95. 26453561 10.1007/s11655-015-2287-y

[17] Wang Y Liu Y Wu Q Rapid and sensitive determination of major active ingredients and toxic components in GinkgoBiloba leaves extract (EGb 761) by a validated UPLC-MS-MS method. J Chromatogr Sci 2017;55:459–64. 28069691 10.1093/chromsci/bmw206

[18] Chen LE Wu F Zhao A Protection efficacy of the extract of Ginkgo biloba against the learning and memory damage of rats under repeated high sustained +Gz exposure. Evid Based Complement Alternat Med 2016;2016:6320586. 27069491 10.1155/2016/6320586 PMC4812286

[19] Lu Q Zou WZ Ji XJ Ethanolic Ginkgo biloba leaf extract prevents renal fibrosis through Akt/mTOR signaling in diabetic nephropathy. Phytomedicine 2015;22:1071–8. 26547529 10.1016/j.phymed.2015.08.010

[20] Zhou X Qi Y Chen T Long-term pre-treatment of antioxidant Ginkgo biloba extract EGb-761 attenuates cerebral-ischemia-induced neuronal damage in aged mice. Biomed Pharmacother 2017;85:256–63. 27863840 10.1016/j.biopha.2016.11.013

[21] Zhu Z Zhou X He B Ginkgo biloba extract (EGb 761) promotes peripheral nerve regeneration and neovascularization after acellular nerve allografts in a rat model. Cell Mol Neurobiol 2015;35:273–82. 25319407 10.1007/s10571-014-0122-1 PMC11486258

[22] Zhang HF Huang LB Zhong YB An Overview of systematic reviews of Ginkgo biloba Extracts for mild cognitive impairment and dementia. Front Aging Neurosci 2016;8:276. 27999539 10.3389/fnagi.2016.00276 PMC5138224

[23] Liu X Hao W Qin Y Long-term treatment with Ginkgo biloba extract EGb 761 improves symptoms and pathology in a transgenic mouse model of Alzheimer's disease. Brain Behav Immun 2015;46:121–31. 25637484 10.1016/j.bbi.2015.01.011

[24] Yancheva S Ihl R Nikolova G Ginkgo biloba extract EGb 761(R), donepezil or both combined in the treatment of Alzheimer's disease with neuropsychiatric features: a randomised, double-blind, exploratory trial. Aging Ment Health 2009;13:183–90. 19347685 10.1080/13607860902749057

[25] Chong ZZ S100B raises the alert in subarachnoid hemorrhage. Rev Neurosci 2016;27:745–59. 27442365 10.1515/revneuro-2016-0021

[26] Deng H Kahlon RS Mohite S Elevated plasma S100B, psychotic symptoms, and cognition in schizophrenia. Psychiatr Q 2018;89:53–60. 28435992 10.1007/s11126-017-9514-y

[27] Dong N Diao Y Ding M The effects of 7-nitroindazole on serum neuron-specific enolase and astroglia-derived protein (S100beta) levels after traumatic brain injury. Exp Ther Med 2017;13:3183–8. 28587392 10.3892/etm.2017.4411 PMC5450618

[28] Bluhm B Laffer B Hirnet D Normal cerebellar development in S100B-deficient mice. Cerebellum 2015;14:119–27. 25342137 10.1007/s12311-014-0606-z

[29] Bembea MM Rizkalla N Freedy J Plasma biomarkers of brain injury as diagnostic tools and outcome predictors after extracorporeal membrane oxygenation. Crit Care Med 2015;43:2202–11. 26082978 10.1097/CCM.0000000000001145

[30] Cirillo C Capoccia E Luvone T S100B inhibitor pentamidine attenuates reactive gliosis and reduces neuronal loss in a mouse model of Alzheimer's Disease. Biomed Res Int 2015;2015:508342. 26295040 10.1155/2015/508342 PMC4532807

[31] Dang X Guan L Hu W S100B ranks as a new marker of multiple traumas in patients and may accelerate its development by regulating endothelial cell dysfunction. Int J Clin Exp Pathol 2014;7:3818–26. 25120758 PMC4128993

[32] Buschert J Hohoff C Touma C S100B overexpression increases behavioral and neural plasticity in response to the social environment during adolescence. J Psychiatr Res 2013;47:1791–9. 23972702 10.1016/j.jpsychires.2013.08.001

[33] Bellissima V Visser GH Ververs TF Antenatal maternal antidepressants drugs affect S100B concentrations in fetal-maternal biological fluids. CNS Neurol Disord Drug Targets 2015;14:49–54. 25613501 10.2174/1871527314666150116114033

[34] Luo KR Hong CJ Liou YJ Differential regulation of neurotrophin S100B and BDNF in two rat models of depression. Prog Neuropsychopharmacol Biol Psychiatry 2010;34:1433–9. 20728493 10.1016/j.pnpbp.2010.07.033

[35] Jang BS Kim H Lim SW Serum S100B levels and major depressive disorder: its characteristics and role in antidepressant response. Psychiatry Investig 2008;5:193–8. 10.4306/pi.2008.5.3.193 PMC2796025 20046365

[36] Widiger TA Clark LA Toward DSM-V and the classification of psychopathology. Psychol Bull 2000;126:946–63. 11107884 10.1037/0033-2909.126.6.946

[37] Cao H Keju JU Zhong L Efficacy of hyperbaric oxygen treatment for depression in the convalescent stage following cerebral hemorrhage. Exp Ther Med 2013;5:1609–12. 23837040 10.3892/etm.2013.1043 PMC3702722

[38] Yao X Chen N Ma CH Ginkgo biloba extracts attenuate lipopolysaccharide-induced inflammatory responses in acute lung injury by inhibiting the COX-2 and NF-kappaB pathways. Chin J Nat Med 2015;13:52–8. 25660288 10.1016/S1875-5364(15)60006-1

[39] Yallapragada PR Velaga MK Effect of Ginkgo biloba Extract on Lead-Induced Oxidative Stress in Different Regions of Rat Brain. J Environ Pathol Toxicol Oncol 2015;34:161–73. 26081034 10.1615/jenvironpatholtoxicoloncol.2015013095

[40] Mei N Guo X Ren Z Review of Ginkgo biloba-induced toxicity, from experimental studies to human case reports. J Environ Sci Health C Environ Carcinog Ecotoxicol Rev 2017;35:1–28. 28055331 10.1080/10590501.2016.1278298 PMC6373469

[41] Shah ZA Nada SE Dore S Heme oxygenase 1, beneficial role in permanent ischemic stroke and in Gingko biloba (EGb 761) neuroprotection. Neuroscience 2011;180:248–55. 21334424 10.1016/j.neuroscience.2011.02.031 PMC3070771

[42] Wang Z Zhang J Ren T Targeted metabolomic profiling of cardioprotective effect of Ginkgo biloba L. extract on myocardial ischemia in rats. Phytomedicine 2016;23:621–31. 27161403 10.1016/j.phymed.2016.03.005

[43] Li Y Zhang Y Wen M Ginkgo biloba extract prevents acute myocardial infarction and suppresses the inflammation and apoptosisregulating p38 mitogenactivated protein kinases, nuclear factorkappaB and Bcell lymphoma 2 signaling pathways. Mol Med Rep 2017;16:3657–63. 28713946 10.3892/mmr.2017.6999

[44] Wang C Wang B Ginkgo Biloba extract attenuates oxidative stress and apoptosis in mouse cochlear neural stem cells. Phytother Res 2016;30:774–80. 26799058 10.1002/ptr.5572

[45] Zuo W Advances in the studies of Ginkgo Biloba leaves extract on aging-related diseases. Aging Dis 2017;8:812–26. 29344418 10.14336/AD.2017.0615 PMC5758353

[46] Chen X Hong Y Zheng P Efficacy and safety of extract of Ginkgo biloba as an adjunct therapy in chronic schizophrenia: a systematic review of randomized, double-blind, placebo-controlled studies with meta-analysis. Psychiatry Res 2015;228:121–7. 25980333 10.1016/j.psychres.2015.04.026

[47] Montes P Ruiz-Sanchez E Rojas C Ginkgo biloba extract 761: a review of basic studies and potential clinical use in psychiatric disorders. CNS Neurol Disord Drug Targets 2015;14:132–49. 25642989 10.2174/1871527314666150202151440

[48] Peng Q Yu N Clinical applieation of S100B protein in children with central nervous system infections: a review. Zhonghua Er Ke Za Zhi 2015;53:393–6. 26080676

[49] Yang T Cheng J Yang Y S100B mediates stemness of ovarian cancer stem-like cells through inhibiting p53. Stem Cells 2017;35:325–36. 27501952 10.1002/stem.2472

[50] Wu L Zhou X Xiao Z Functional expression, characterization, and application of human S100B. Oncol Rep 2017;38:2309–16. 28849099 10.3892/or.2017.5922

[51] Wu H Brown EV Acharya NK Age-dependent increase of blood-brain barrier permeability and neuron-binding autoantibodies in S100B knockout mice. Brain Res 2016;1637:154–67. 26907191 10.1016/j.brainres.2016.02.026

[52] Canzobre MC Rios H Pulpar tooth injury induces plastic changes in S100B positive astroglial cells in the trigeminal subnucleus caudalis. Neurosci Lett 2010;470:71–5. 20043976 10.1016/j.neulet.2009.12.060

[53] Stromberg S Björklund MG Asplund A Transcriptional profiling of melanocytes from patients with vitiligo vulgaris. Pigment Cell Melanoma Res 2008;21:162–71. 18426409 10.1111/j.1755-148X.2007.00429.x

[54] Geduldig ET Kellner CH Electroconvulsive therapy in the elderly: new findings in geriatric depression. Curr Psychiatry Rep 2016;18:40. 26909702 10.1007/s11920-016-0674-5

[55] Hummel J Weisbrod C Boesch L AIDE-acute illness and depression in elderly patients. Cognitive behavioral group psychotherapy in geriatric patients with comorbid depression: a randomized, controlled trial. J Am Med Dir Assoc 2017;18:341–9. 27956074 10.1016/j.jamda.2016.10.009

[56] Eyre HA Genomic predictors of remission to antidepressant treatment in geriatric depression using genome-wide expression analyses: a pilot study. Int J Geriatr Psychiatry 2016;31:510–7. 26471432 10.1002/gps.4356 PMC5567872

[57] Kennedy SH Lam RW Mcintyre RS Canadian network for mood and anxiety treatments (CANMAT) 2016 clinical guidelines for the management of adults with major depressive disorder: section 3. pharmacological treatments. Can J Psychiatry 2016;61:540–60. 27486148 10.1177/0706743716659417 PMC4994790

[58] Ising M Lucae S Binder EB A genomewide association study points to multiple loci that predict antidepressant drug treatment outcome in depression. Arch Gen Psychiatry 2009;66:966–75. 19736353 10.1001/archgenpsychiatry.2009.95 PMC4465570

[59] Zong Y Xue Y Zhao Y Depression contributed an unsatisfactory surgery outcome among the posterior decompression of the cervical spondylotic myelopathy patients: a prospective clinical study. Neurol Sci 2014;35:1373–9. 24643580 10.1007/s10072-014-1714-8

[60] Guiard BP Di Giovanni G Central serotonin-2A (5-HT2A) receptor dysfunction in depression and epilepsy: the missing link? Front Pharmacol 2015;6:46. 25852551 10.3389/fphar.2015.00046 PMC4362472

[61] Machado RD The molecular genetics and cellular mechanisms underlying pulmonary arterial hypertension. Scientifica (Cairo) 2012;2012:106576. 24278664 10.6064/2012/106576 PMC3820608

[62] Costa L Novel agonists for serotonin 5-HT7 receptors reverse metabotropic glutamate receptor-mediated long-term depression in the hippocampus of wild-type and Fmr1 KO mice, a model of Fragile X Syndrome. Front Behav Neurosci 2015;9:65. 25814945 10.3389/fnbeh.2015.00065 PMC4357247

[63] Rapin JRZM Drieu K In vitro and in vivo effects of an extract of Ginkgo biloba (EGb 761), ginkgolide B, and bilobalide on apoptosis in primary cultures of rat hippocampal neurons. Drug Development Research 2015;45:23–9.

[64] Sibolt G Curtze S Melkas S Post-stroke depression and depression-executive dysfunction syndrome are associated with recurrence of ischaemic stroke. Cerebrovasc Dis 2013;36:336–43. 24193249 10.1159/000355145

[65] Lohner V Brookes RL Hollocks MJ Apathy, but not depression, is associated with executive dysfunction in cerebral small vessel disease. PLoS One 2017;12:e0176943. 28493898 10.1371/journal.pone.0176943 PMC5426624

